# Regression of Hypervascular Nodules in a Patient with Wilson's Disease Awaiting Liver Transplantation

**DOI:** 10.1155/2009/597371

**Published:** 2009-11-11

**Authors:** Alcindo Pissaia, Hervé Gouya, Olivier Scatton, Filoména Conti, Yvon Calmus

**Affiliations:** Liver Transplant Unit and Radiology Department, Hôpital Cochin, Université Paris Descartes, 75679 Paris, France

## Abstract

This paper describes the regressive course over one year of hypervascular nodules in a patient with Wilson's disease. CT revealed multiple, enhancing nodules (up to 3 cm in diameter) detected in the liver in the early arterial phase after the administration of intravenous contrast material. Most of these nodules became isodense in the portal venous phase. After one year of efficient therapy combining d-penicillamine and zinc acetate, most of the nodules had disappeared, while the liver contours had become more regular. To our knowledge, the regression of large hypervascular nodules has not previously been reported in patients with Wilson's disease.

## 1. Introduction

Wilson's disease (WD) is an autosomal recessive disorder characterized by defective copper excretion. WD occurs worldwide with an average prevalence of 10–30 affected individuals per million of the population. The gene that is abnormal in WD, *ATP7B*, encodes a metal-transporting ATPase and was identified in 1993 [1, 2]. An absent or reduced function of ATP7B protein leads to a decreased hepatocellular excretion of copper into bile, resulting in hepatic copper accumulation and injury, an increased release of copper into the bloodstream and its deposition in various organs, notably the brain, kidneys, and cornea. A failure to incorporate copper into ceruloplasmin results in lower blood levels of ceruloplasmin because of the reduced half-life of the apoprotein.

The clinical features of WD include hepatic abnormalities, neurological defects (extrapyramidal features, seizures), and psychiatric symptoms which are markedly heterogeneous, even among patients with the same mutations [3]. WD often presents with prominent liver disease in children and young adults. However, hepatocellular carcinoma is a rare complication of WD.

Liver transplantation is indicated in the event of decompensated liver disease unresponsive to medical therapy and in patients who present with fulminant hepatic failure [4, 5]. One-year survival following liver transplantation ranges from 79% to 87%, and long term survival is excellent. 

This paper describes the regressive course over one year of hypervascular nodules in a patient with Wilson's disease.

## 2. Case Report

A 21-year-old man from Saudi Arabia, who had experienced symptoms for one year prior to the diagnosis of WD and presented with cirrhosis possibly complicated by hepatocellular carcinoma, was referred to our center with a view to liver transplantation. 

On admission, his vital signs were normal and his consciousness was clear. Physical examination revealed jaundice, moderate ascites, splenomegaly, and extrapyramidal rigidity. Levels of total bilirubin (46 *μ*mol/L, *N* < 17), direct bilirubin (17 *μ*mol/L, *N* < 2), aspartate aminotransferase (77 IU/L, *N* < 40), and alanine aminotransferase (57 IU/L, *N* < 40) were high. Gamma glutamyl transpeptidase levels (50 IU/L, *N* < 80) were within the normal range. The prothrombin rate (64%, INR 1.38) and albumin levels (29 g/L) were both low. Markers of hepatitis virus and autoimmune hepatitis were all negative. Serum copper levels were normal (13 *μ*mol/L) and serum ceruloplasmin levels were low (0.08 g/L, *N* 0.17–0.70), while urinary copper levels were high (2.2 *μ*mol/24 hours). The alphafetoprotein value was 16.7 *μ*g/L.

**Figure 1:**
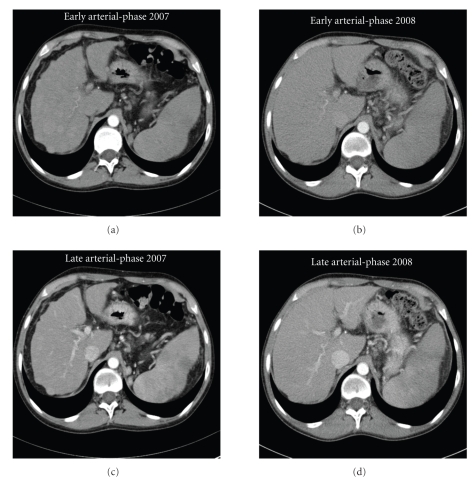
Arterial-phase axial CT images following the administration of contrast medium demonstrate multiple hyperdense nodules at the early arterial phase. Nodules are not visible on unenhanced images.

Abdominal ultrasonography (US) and computed tomography (CT) scans revealed liver cirrhosis and splenomegaly. US showed that the liver contours were irregular and its echogenicity was increased, with multiple, hypo- and hyperechoic nodules. CT scans were performed using a 64-detector dual-source DE scanner (Somatom DefiSiemens Medical Solutions, Forchheim, Germany), with a four-phase technique that included the acquisition of nonenhanced images and a three-phase intravenous contrast material-enhanced CT during the hepatic arterial, portal venous, and delayed phases. Scanning delays were 20–28 seconds for arterial phase imaging, 60–70 seconds for portal venous phase imaging, and 300 seconds for delayed imaging phase. The CT scans revealed a liver of reduced volume with marked contour irregularity consistent with chronic liver disease. Multiple hypervascular nodules (the largest being 3 cm in diameter) were depicted at the arterial phase under contrast-enhanced CT (Figure 1). All nodules became isoattenuating or hypoattenuating to the surrounding liver parenchyma during either the portal venous phase or the delayed phase.

Slit-lamp examination revealed Kayser-Fleischer rings on the patient's cornea. Cerebral MR imaging was performed using a 1.5 T system (Signa, GE Medical Systems, Milwaukee, Wis.) equipped with the standard head coil. MR sequences included transverse, unenhanced T1-weighted images (TR/TE, 580/20; 5 mm section thickness; interslice gap, 1.5 mm; field of view, 24 × 18), transverse T2-weighted fast spin-echo images (3.800/98; 5 mm section thickness; interslice gap, 1.5 mm; field of view, 24 × 24), transverse FLAIR sequences (9.802/156, 5 mm section thickness; interslice gap, 1.5 mm; field of view, 24 × 24), diffusion-weighted images, and 3D T1-weighted after IV contrast injection (gadopentetate dimeglumine, 0.1 mmol/kg) with multiplanar reconstructions. T1-weighted, T2-weighted, diffusion-weighted, and Flair images revealed increased signal intensity in the putamen. No abnormalities were found in the thalami, caudate and globus pallidus, cortical white matter, the pons, the midbrain, or cerebellum. Since no other recognized case was available in the patient's family, a direct mutation analysis was performed and revealed a compound heterozygous for WD (Ser744Pro substitution and 4193 delc deletion).

D-penicillamine (Trolovol, Dexo) was initiated at 300 mg/d and then increased to 1200 mg/d in 2 divided intakes. Pyridoxine (35 mg) (vitamin B1B6, Bayer) and zinc acetate dihydrate (Wilzin, Orphan Europe) (100 mg/d) were added. Tolerance was excellent, without any significant modifications to the blood count and creatinine levels, and without significant proteinuria or leucocyturia. After 30 days of treatment, 24-hour urinary copper excretion was 37 *μ*mol. 

One year after treatment initiation, physical examination revealed neither jaundice or ascites. Splenomegaly and extrapyramidal rigidity persisted. Total bilirubin (26 IU/L), direct bilirubin (8 IU/L), aspartate aminotransferase (42 IU/L), alanine aminotransferase (41 IU/L), gamma glutamyl transpeptidase (38 IU/L), prothrombin rate (72%), and albumin (32 g/L) values had all improved. Three-phase abdominal CT revealed increased hepatic volume and reduced contour irregularity. At the arterial phase, a dramatic reduction in the number and size of nodules (the largest being 1 cm in diameter) and hyperdense nodules was observed (Figure 1). All these nodules became isodense during the portal venous phase. 

## 3. Discussion

Imaging findings in cirrhotic patients include an increased echogenicity of the liver, the irregularity of contours, and atrophied lobes or segments. During one study of 33 WD patients, US examination revealed disorders affecting the hepatic echotexture (29 cases), changes to splenic dimensions (21 cases), liver shrinkage (10 cases), cholelithiasis (8 cases), and ascites (1 case) [6]. Disorders affecting liver echotexture displayed different patterns, ranging from slight to severe changes in hepatic echogenicity, associated with anatomical distortions of the liver, such as alterations in outline and a decrease in dimensions [6]. These findings are indistinguishable from those of cirrhosis due to other causes. Multiple round foci of decreased echogenicity resembling metastatic liver disease [7] and hyperechogenic nodules [8] have also been reported on rare occasions. 

It is generally accepted that CT of the liver offers little information regarding the diagnosis or management of WD. Nodularity in the liver has only been observed in a few WD patients [9, 10]. Multiple hyperdense areas in the liver on unenhanced CT images were reported in a 2-year-old child [11]. These lesions were hyperintense on T1-weighted, and hypointense on T2-weighted, MRI images. In a 12-year-old child, nodules were only detectable after the administration of contrast material for CT [8]. Large hypervascular nodules may be dysplastic or neoplastic lesions. More rarely, they correspond to regenerative nodules. In the series of Akhan, the arterial enhancing nodules were secondary to dysplastic changes [8], and the lesions were best appreciated on early arterial phase images following the administration of intravenous contrast medium. Low-grade dysplastic nodules are normally supplied by the portal vein and therefore are isointense to liver during the arterial phase. The signal intensity characteristics of some high-grade dysplastic nodules, which receive increasing supply from the hepatic artery, may overlap with those of hepatocellular carcinoma [12]. During another study, arterial phase enhancement of nodular lesions in a cirrhotic liver during CT was interpreted as hepatocellular carcinoma [13]. Indeed, size larger than 2 cm and rapid interval growth in terms of arterial hypervascularity are the usual criteria favoring malignancy [12]. However, most patient series have shown that liver carcinogenesis is reduced in WD when compared to other causes of cirrhosis [14]. The blood supply of regenerative nodules is largely dependent from the portal vein, with minimal contribution from the hepatic artery. This vascular supply dynamic explains why there is no enhancement during the hepatic arterial phase on CT or MR images, although arterial phase enhancement in regenerative nodules has been described and can be mistaken for hepatocellular carcinoma [15]. Liver biopsy is usually performed to distinguish between dysplastic or neoplastic nodules and regenerative nodules, although sampling errors are frequent.

In our case, the liver biopsy was refused by the patient. A diagnosis of regenerative or dysplastic nodules was proposed, and the patient was regularly monitored while on the waiting list for liver transplantation, without liver biopsy. The regression of these nodules after only one year of efficient therapy and improvements in liver function parameters led to the patient's removal from the waiting list. 

A reduction in copper accumulation under d-penicillamine therapy may reduce the inflammatory process and, in the long term, lead to a regression of fibrosis. In addition, zinc salts have been shown to inhibit the production of oxygen-activated species and reduce lipid peroxidation [16]. We can thus hypothesize that combined therapy with d-penicillamine and zinc salts acted synergistically to improve the fibrogenic and dysplastic processes. Alternatively, changes in hemodynamics of the patient due to the successful therapy are possible. Finally, it can not be ruled out that attenuation of contrast on the second CT scan, in comparison with the first one, may play a role in the differences observed.
